# Randomized Clinical Trial of Electrostimulation Therapies as an Adjuvant for the Treatment of Temporomandibular Disorders

**DOI:** 10.3390/dj12080273

**Published:** 2024-08-22

**Authors:** Claudia I. Rodríguez, Fernando Angeles, Socorro A. Borges, Eduardo Llamosas, Julio Morales

**Affiliations:** 1 Faculty of Dentistry, National Autonomous University of Mexico, Mexico City 04510, Mexico; anfer522@unam.mx (F.A.); aborges@unam.mx (S.A.B.); juliomorales@fo.odonto.unam.mx (J.M.); 2 Faculty of Higher Studies Iztacala, National Autonomous University of Mexico, Mexico City 04510, Mexico; llamosas@unam.mx

**Keywords:** clinical trial, temporomandibular disorders, electrostimulation therapies, occlusal splint, electromyography, neuromuscular electrical activity

## Abstract

We carried out a comparison of the neuromuscular and clinical effects produced by electrostimulation therapies, as an adjuvant to the use of occlusal splints (removable muscle relaxation apparatus) in patients with temporomandibular disorders In this simple randomized clinical trial, 91 patients were randomly divided into three groups. Group A (GA) received transcutaneous electrostimulation therapy and an occlusal splint, Group B (GB) received percutaneous electrostimulation therapy and an occlusal splint, and Group C (GC) received an occlusal splint. The neuromuscular activity, as well as the signs and symptoms of each patient, were evaluated every week throughout the treatment (T0 = baseline; T1 = 7 days; T2 = 14 days; T3 = 21 days; T4 = 28 days; and T5 = 35 days). Pain was measured with a visual analog scale, and neuromuscular electrical activity was determined by the root mean square of the masseter muscles through the use of a UNAM-CINVESTAV 1.2 electromyograph. Comparisons were made using ANOVA for repeated measures (*p*-value = 0.05). The comparison between the groups determined that muscle fatigue (*p*-value = 0.001), joint pain (*p*-value = 0.009), and muscle pain (*p*-value = 0.003) decreased to a greater extent, and in the short term for the group treated with transcutaneous electrostimulation therapy as an adjuvant to the use of the occlusal splint. The comparison between the groups determined that muscle fatigue (*p*-value = 0.001), joint pain (*p*-value = 0.009), and muscle pain (*p*-value = 0.003) decreased to a greater extent and in a shorter term in the GA (calculation therapy, transcutaneous electrostimulation) and GB (occlusal splint). Transcutaneous electrostimulation is a feasible and faster alternative that was accepted by most of the patients for treating temporomandibular disorders.

## 1. Introduction

Temporomandibular disorders (TMDs) are alterations in masticatory function [1]. They are defined as a set of clinical problems affecting the masticatory muscles, the temporomandibular joint (TMJ), and the associated tissues [2].

TMD is the main cause of maxillofacial pain of non-dental origin. Epidemiological data report that TMD is the second most common musculoskeletal disorder causing pain and disability [3,4]. It has highly variable symptoms, which can affect daily living activities [5,6]. TMDs mainly affect the population between 20 and 40 years old; the prevalence can vary between 40% and 60%, with an estimated 10% of cases being severe [7]. The high prevalence of TMDs, the lack of epidemiological indicators, and the shortage of treatment guidelines make TMDs a relevant public health problem [3]. TMDs are the second most common musculoskeletal disorder causing pain and disability [3,4]. TMDs are identified as the main cause of myofascial pain of non-dental origin. They cause pain in the muscles that control maxillary and mandibular function, the neck, shoulders, head, face, TMJ area, and back. They have highly variable symptoms, which can affect daily living activities [2]. TMDs mainly affect the population between 20 and 40 years old; the prevalence can vary between 40% and 60%, with an estimated 10% of cases being severe.

TMD has a multifactorial etiology because the psychological factors lead to altered muscle activity, increasing muscle tension and pain tolerance levels, causing signs and symptoms of TMD.

The Diagnostic Criteria for Temporomandibular Disorders (DC/TMD) offers a standardized system to evaluate temporomandibular dysfunction (TMD), uses clinical examination methods to obtain clinical signs of TMD (Axis I), and the evaluation of the behavioral, psychological, and psychosocial aspects (Axis II). Axis I allows for classifying the diagnosis of TMD; according to its etiology, it could be divided into Group I: muscular disorders (including myofascial pain with and without limitation of mouth opening); Group II: disc displacement with or without reduction and limitation of mouth opening; Group III: arthralgia, arthritis, and osteoarthritis) [4].

The main goal of TMD treatment is to restore function; the clinical challenges are the eradication of pain, a reduction in the noises in the joint, and the recovery of normal jaw movement. There are many types of treatments for TMD, surgical or non-surgical: patient education, behavioral therapy, psychotherapy, pharmacotherapy, physical therapy, mechanical therapy, occlusal therapy, and surgical treatments.

Pharmacotherapy includes the use of analgesics and non-steroidal anti-inflammatory drugs (NSAIDs) (aspirin, ibuprofen, paracetamol, ketorolac, ketoprofen, piroxican, naproxen, phosphosal, and diclofenac); narcotics (codeine and propoxyphene); muscle relaxants (carisoprodol, methocarbamol, and meprobamate); tranquilizers (benzodiazepines, phenotyzines, and diazepam); and tricyclic antidepressants (amitriptyline and imipramine) [8,9].

Nowadays, there are different types of treatments, less invasive and more relevant, first-line, such as physiotherapy and behavioral treatment and others such as the use of occlusal splints, pharmacological treatments, infiltration, and surgery. The occlusal splint is a rigid removable appliance that covers all the teeth of the upper arch, made of thermoplastic material and an acrylic reline. Splints are the first-choice treatment for a conservative approach because they collaborate with muscle relaxation, helping the positioning of the condyle and mitigating pain [3]. Occlusal splints aim to generate functional stability in the jaw [4,8]. Occlusal splints can be classified by function (for muscle relaxation, mandibular repositioners, reducing planes, distractors, and protectors), therapeutic purpose (with and without programmed therapeutic modification of the condylar position), coverage (partial or total), and hardness (rigid, semi-rigid, and resistant).

The success of occlusal splints in the treatment of patients with TMD is widely documented in the dental literature [9,10,11,12,13]. The mode of operation of occlusal splints involves the following important aspects: (a) change in position of the condyles and discs; (b) increase in vertical dimension, producing a change in muscular response; (c) perception of symptoms—patients manifest self-knowledge of functional and parafunctional behavior, reducing bad habits and frequency; (d) placebo effect; and (e) occlusal stabilization—temporary elimination of the first occlusal contacts, reducing isometric muscle tensions [14]. It remains possible that the short-term effect of any device or appliance is associated with muscle fibers and spindle adaptation to changes in jaw position, or to tongue position and airway patency [15]. Occlusal splints or stabilization splints are indicated for 3 to 4 months, with an average daily use of more than 12 h. It is important to mention that occlusal splints are a means to reinforce the patient’s self-care (initial phase of treatment), supported with psychotherapy, behavioral education, physiotherapy, and exercise routines at home, all with the aim of developing the patient’s ability to understand the principle and implications of this treatment [16]. Despite the advantages that occlusal splints provide (aesthetics, comfort, stability, and functionality), it is relevant, during treatment, to provide effective follow-up to patient because worsening of symptoms requires immediate re-evaluation to provide explanations, corrections, or necessary adjustments, but also a reassessment of the diagnosis. “The occlusal splint remains an element of a global therapeutic solution”. It is necessary to optimize treatment for TMD with conservative and multidisciplinary approach [17,18,19]; a randomized clinical trial compared the effectiveness of low-level laser therapy and laser acupuncture therapy in patients with temporomandibular disorders [11]. The Academy of Orofacial Pain recommends analgesic therapies with the use of occlusal splints to reduce pain, restore function, and decrease some other factors that trigger TMDs, such as stress and anxiety [9].

Clinical studies have shown the feasibility of significantly reducing myofascial pain by electrical stimulation, which has analgesic and anti-inflammatory effects on the muscles [12]. For the treatment of TMD, electrostimulation can be applied through two major routes: transcutaneous electrical nerve stimulation (TENS) and percutaneous neuromodulation therapy (PNT) [12].

Transcutaneous electrostimulation refers to the application of an electric current to the skin via surface electrodes; it is supported by the Food and Drug Administration (FDA) [13] as an effective method for the treatment of pain [14]. The FDA recommends its use under the supervision of a healthcare professional [15]. The mechanism of action of transcutaneous electrostimulation consists of activating the nerve endings of the skin [16], producing changes at the brain and the cortical level, altering the neural axis [17].

Percutaneous electrostimulation, also called electroacupuncture, acts by administering a direct electrical current into the deep muscle tissues, and stimulates the thick afferent fibers (sensitive fibers) located at the origin of pain [18,19].

In odontology, different therapies are applied, based on the same principle, and it has been observed that the degree of activation determines the magnitude of the effect on the organ’s functions—for example, inhibition of pain [20]. Studies differ on the location of the area to be stimulated, how long the stimulus should last, and the amount and frequency of therapy [21].

Electromyography (EMG) is a diagnostic procedure used to evaluate the health of muscles and the nerve cells that control them (motor neurons). Electromyography results may reveal nerve dysfunction, muscle dysfunction, or problems with the transmission of signals from nerves to muscles. Motor neurons transmit electrical signals that cause muscles contraction. In electromyography, surface electrodes are used to transmit these signals and thus measure the speed and intensity of the signals that travel between the muscles under evaluation [22].

EMG is a very low-risk procedure, and complications are unusual. There is a low risk of hemorrhage, infection, and nerve injury when recording with needles, where a needle-shaped electrode is inserted [23]. Methods for processing and analyzing electromyographic signals can be found in [24].

Therefore, the objective of this study was to compare the neuromuscular electrical activity (RMS) of the masseter muscles in three groups of patients with different treatments for TMD (Group A (GA): transcutaneous electrostimulation and a splint; Group B (GB): percutaneous electrostimulation and a splint; Group C (GC): occlusal splint) via six electromyographic recordings scheduled weekly with patients of the Physiology Laboratory of the Division of Postgraduate Studies and Research (DEPeI) at UNAM, admitted during the period from August to December 2021.

## 2. Materials and Methods

### 2.1. Trial Design

A simple randomized clinical trial was conducted, with the design and guidelines reported, according to the recommendations of CONSORT [16]. This study was conducted at the physiology laboratory of the Division of Postgraduate Studies and Research (DEPeI) of the National Autonomous University of Mexico (UNAM). This study was approved by the Research and Ethics Committee of the Faculty of Dentistry of the National Autonomous University of Mexico (CIE/0508/02/2020, 8 February 2020). All participants signed an informed consent form.

The physiology laboratory of the DEPeI UNAM assists approximately 900 patients per year, of whom 168 patients are referred for the first time from other specialty clinics of the DEPeI UNAM. About 100 appointments are scheduled per month; most of the consultations are for monitoring treatment through the adjustment of occlusal splints and electromyographic recordings. Patients received by the physiology laboratory are referred by the Reception and Diagnosis Clinic (Postgraduate), the specialty clinics of the DEPeI, the stomatological services of hospitals, and private practices, among others.

### 2.2. Participants

Our study population included patients with TMD attending to the physiology laboratory of the Division of Graduate Studies and Research (DEPeI) UNAM.

The study population comprised patients with temporomandibular disorder referred by the DEPeI Reception and Diagnosis Clinic during the period from August to December 2021. The eligible population consisted of those who met the following inclusion criteria: patients between 18 and 60 years old, who had not undergone previous TMD treatment; with permanent dentition; with malocclusion; patients with any signs or symptoms of TMDs (altered mandibular function, joint sounds, or muscle or joint pain); patients with prosthetic treatments; and patients with bruxism. 

On the other hand, the following were excluded: patients with periodontal problems; those undergoing orthodontic or orthopedic treatment; those with apparent neurological or sensory disabilities; those with bleeding disorders; those with cardiac pathologies; patients with epilepsy, facial wounds, or severe acne; patients with neurological or muscular disorders; patients who were allergic to metal; patients with acute infections or inflammatory processes; pregnant patients; patients with a history of joint surgery; patients with malignancies; patients with degenerative bone diseases; patients with fibromyalgia; patients with mental disorders; those with altered orofacial motor function; those with uncontrolled systemic diseases; those with severe oral manifestations; and those with psychological or psychiatric disorders that would influence the outcome. This information was anamnestically evaluated by physicians and was taken from the patient registry. Once these criteria were applied, the randomized sample included 91 patients.

### 2.3. Interventions

In total, 91 patients received treatment for TMD. Of these, 31 patients were treated with transcutaneous electrostimulation and an occlusal splint, 27 patients were treated with percutaneous electrostimulation and an occlusal splint, and 33 patients used an occlusal splint only.

The principal investigator, a professor and specialist in orthodontics with 5 years of experience, performed the clinical examinations and treatments. First, the diagnosis of TMD was made using the Diagnostic Criteria for Temporomandibular Disorders (DC/TMD). The principal investigator was standardized in the University of the Republic of Uruguay for the clinical measure regarding Axis1 of the DC/TMD. 

The signs and symptoms of TMD were recorded. A digital algometer was used for assessing pain, in which 1.5 kg/cm^2^ of force was applied to the masseter muscles and the area of the TMJ. The pain reported by each patient was measured with a visual analog scale (VAS). 

The level of anxiety was evaluated with the Beck anxiety inventory (BAI), and stress with the perceived stress scale (PSS-14). The Beck anxiety inventory (BAI) scale is a self-report measure of anxiety with 21 items; the total score is calculated through the sum of the 21 items: low anxiety (0–21), moderate anxiety (22–35), and potentially concerning levels of anxiety (>36). Reliability: internal consistency for the BAI (Cronbach’s α = 0.92); test–retest reliability (1 week) for the BAI = 0.75 [25]. 

The Beck anxiety inventory (BAI) scale was moderately correlated with the revised Hamilton anxiety rating scale, and mildly correlated with the Hamilton depression rating scale.

Both are self-reported measures that assesses the degree to which the respondent has perceived situations in his/her life within the past month.

The PSS is a five-point scale (1  =  strongly disagree; 2  =  disagree; 3  =  neutral; 4  =  agree; 5  =  strongly agree), with a two-factor structure of perceived stress (PS) and perceived coping (PC), which evaluates if a person’s life is perceived as unpredictable, uncontrollable, or overloaded [26].

Six weekly appointments for treatment were scheduled for each patient in the three groups. In each session, the same evaluator (principal investigator) evaluated the signs and symptoms of TMD (muscle pain, joint pain, presence of joint sounds, and an open mouth), electromyographic recordings, adjustment of the occlusal splint, and application of electrostimulation therapy.

**Figure 1:**
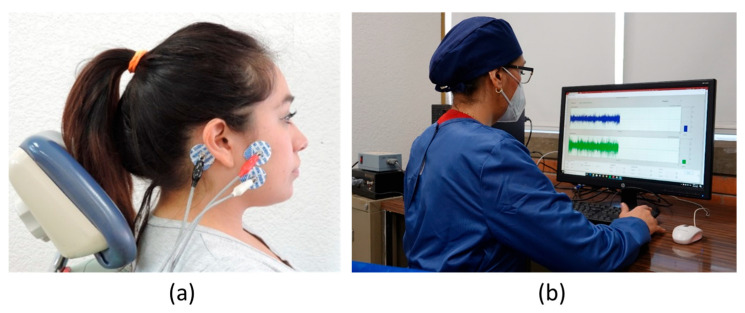
Electromyographic recording. (**a**) Placement of surface electrodes over the origin and insertion of the masseter muscles; (**b**) electromyographic recording in maximum voluntary contraction for 30 s, with Electromyograph 1.2 UNAM-CINVESTAV^®^.

The clinical electromyographic recording method was standardized with intraoperative and interoperative recording tests, and was performed using intraclass correlation coefficients, *p*-value (>0–85). The electromyographic recordings were taken with the Electromyograph 1.2 UNAM-CINVESTAV^®^ recording system (hardware and software). Neuromuscular electrical activity was determined by root mean square (RMS) analysis. For the recording, three electrodes were placed (at the muscle’s insertion, the muscle’s origin, and the retroauricular area) (Figure 1). The recording was made at maximum intercuspation for 30 s; the action potential expressed by the electrical energy was recorded in microvolts per second (μV/s).

The designs of indications for the occlusal splints were the same for all participants. The splints were placed at the physiology laboratory of the DEPeI, UNAM. The indicated use was for 24 h a day (it could be removed to eat and to brush teeth). The splints were made from a vacuum thermoplastic machine, placing a 0.060″-gauge soft acetate sheet, followed by a 0.080″-gauge rigid acetate. Under clinical conditions, a self-curing acrylic rebase was placed on the occlusal surface of the splint, with the aim of achieving a homogeneous occlusal bite. The dimensions of the splint (interdental disocclusion) were verified, taking care that it did not interfere with any mandibular movement. 

**Figure 2:**
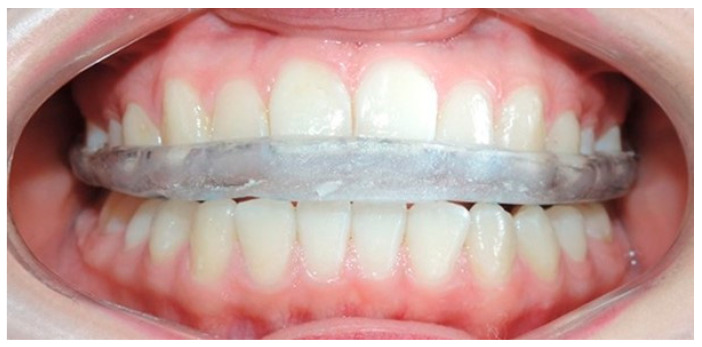
Occlusal splint.

**Figure 3:**
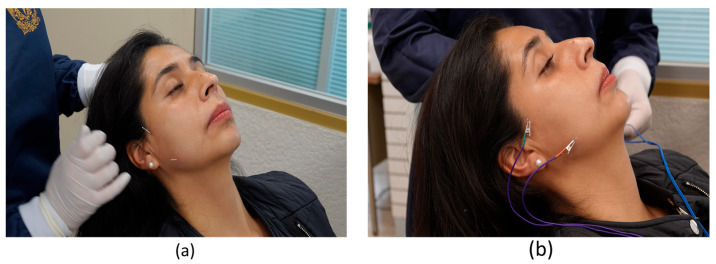
Percutaneous electrostimulation therapy. (**a**) Insertion of needles into the masseter muscle; (**b**) application of electrical stimulation with electroacupuncture equipment.

Group A, as an adjuvant to the use of splint (Figure 2), received percutaneous electrostimulation therapy with disposable sterile steel acupuncture needles measuring 0.25 × 13 mm (brand AcuBEST, FDA 510K) and portable electrostimulator equipment (KWD-808). For placing the needles, the superficial area of the masseter muscle was cleaned with alcohol. The puncture was performed by taking the needle by the body (not by the handle), holding the lower part between the thumb and index finger of the right hand, letting the tip of the needle pass, and directing the needle to the acupuncture point to insert it quickly (penetrating 5 mm) (Figure 3).

Two needles were placed in each masseter muscle, following the Practical Recommendations for the Treatment of Temporomandibular Disorders. The area to be punctured was the origin of the masseter muscle or the superficial area of the mandibular condyle anterior to the atrial tragus, approximately 1 cm from where they were connected to the KWD-808 electroacupuncture machine with the positive pole (red) on the origin of the masseter muscle, and the negative pole (black color) on the insertion of the masseter muscle. The principal investigator was qualified to apply traditional Chinese medicine methods as specified by the World Health Organization.

A square or rectangular wave current was transmitted with two frequency bands (low, 210 Hz; high, 10,140 Hz.), a wave amplitude of 3050 volts, a pulse duration of 40,100 msec, and an output intensity of 0.70 (for 100 Hz). The time of each treatment was 20 min.

**Figure 4:**
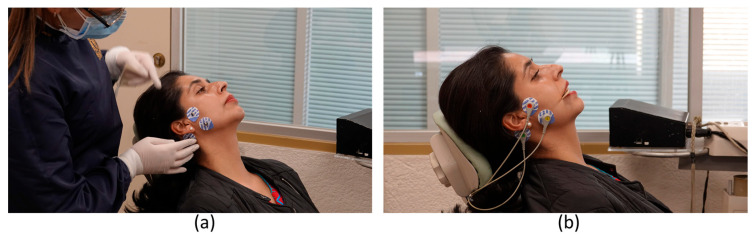
Transcutaneous electrostimulation therapy. (**a**) Placing electrodes on the masseter muscle; (**b**) application of electrical stimulation with electroacupuncture equipment.

For patients in Group B, transcutaneous electrostimulation therapy (Figure 4) was used via a Kendall^®^ MediTrace 100, (pediatric) conductive adhesive ECG electrodes (2.4 cm in diameter), and KWD-808 portable electrostimulatory equipment. This therapy used the same parameters as percutaneous electrostimulation therapy. 

### 2.4. Results

The main outcome was the neuromuscular electrical activity determined by RMS analysis of the electromyographic recording of the masseter muscles, as well as the signs and symptoms of TMD (muscle pain, joint pain, the presence of joint sounds, and an open mouth). Six measures were taken, one every week. 

### 2.5. Sample Size

The sample size was calculated to compare the means of two or more groups via single-factor analysis of variance (ANOVA). We assumed that there were three groups to evaluate, an alpha value of 0.05, a confidence value of 1.96, a power of 1.28, an intergroup variance of 74 μV, and an error of 5.56 μV (Cohen difference) in the RMS evaluation. With this, the minimum sample size per group was 25 patients. The sample size estimated for our study included an assumption of 10% attrition each week, yielding a total sample size of 84 patients (28 patients in each group). 

### 2.6. Randomization

#### 2.6.1. Balanced Block Randomization

The random sequence was generated by Minitab software vesion 3.6.1, using the SAMPLE function.

#### 2.6.2. Concealment of Allocation

The assignment of the subjects to the different groups of clinical procedures (A, B, or C) was performed by an external researcher, who applied a simple random sequence.

#### 2.6.3. Implementation

Personnel external to the described above procedures enclosed the coding sequence in envelopes that were delivered to each patient. The principal investigator opened the envelope during the first appointment to apply the appropriate type of treatment. 

### 2.7. Blinding

It was not possible to blind the investigator who applied the TMD treatments in all three groups. The patients were informed about which group they belonged to.

### 2.8. Statistical Methods

The main analysis had an intention-to-treat approach. This included all randomized patients. First, a descriptive analysis of the main clinical characteristics of the population to be studied was performed, reporting the mean and standard deviation; or the median and its interquartile range, according to whether it had a normal distribution; or as the number and percentage. To identify changes in the electromyographic and clinical variables over time during the TMD treatment, multivariate models (Hox, 2002) (White, 1980) were applied. Since each subject was evaluated in five separate instances every 7 days, the models were adjusted to describe the effect of time (T0 = baseline measurement; T1 = 7 days; T2 = 14 days; T3 = 21 days; T4 = 28 days; and T5 = 35 days), with the explanatory variables compared with the baseline. A bivariate analysis was performed to determine the explanatory variables that were candidates for inclusion in the multivariate analysis, using the coefficient criteria associated with the significant explanatory variable at 5%.

Subsequently, a functional data analysis [27] was performed to obtain an average curve for each instance of the experiment, stratified by time and by treatment group.

A one-way ANOVA was performed to compare the electromyographic and clinical effects of each type of treatment, applied in the three groups. The significance level was set at *p* = 0.05. All tests were performed using Stata 15 statistical software. 

## 3. Results

A sample of 91 patients with TMD was treated: 31 patients were treated with transcutaneous electrostimulation and an occlusal splint, 27 patients were treated with percutaneous electrostimulation and an occlusal splint, and 33 patients used an occlusal splint only. The main outcome was the neuromuscular electrical activity determined by RMS analysis of the electromyographic recording of the masseter muscles, as well as the signs and symptoms of TMD (muscle pain, joint pain, presence of joint sounds, and an open mouth). Six measurements were taken, one each week.

### 3.1. Flow of Participants

**Figure 5:**
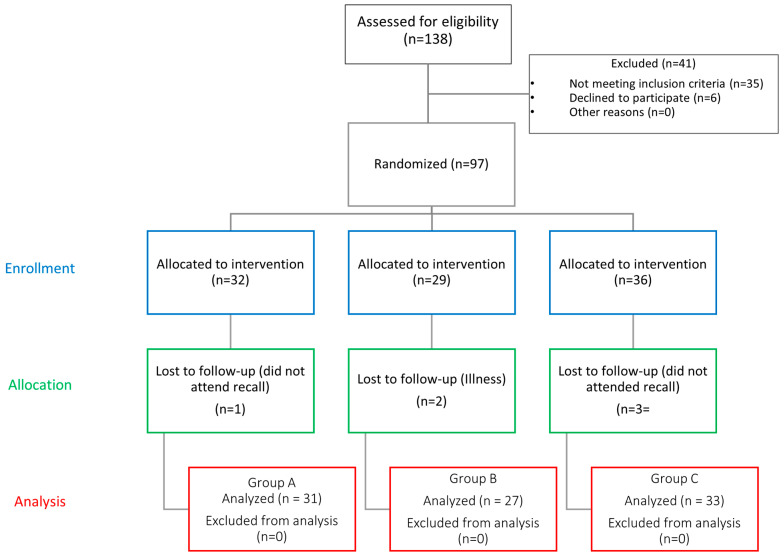
Flowchart of patients in this study.

The flow of participants is described in Figure 5 Group A included 31 patients treated with percutaneous electrostimulation therapy and occlusal splints; Group B included 27 patients treated with transcutaneous electrostimulation therapy and occlusal splints; Group C included 33 patients treated with occlusal splints only.

### 3.2. Recruitment

Patients were recruited between January and June 2021. Initial data were collected from March to September 2021. Of the 138 patients examined, 97 met the inclusion criteria. These were invited to participate, and all accepted.

### 3.3. Reference Data

The baseline demographic data of patients who completed the treatment are shown in Table 1.

### 3.4. Numbers Analyzed

Data from 38 patients were analyzed: 17 in the conventional group and 21 in the simplified group.

### 3.5. Results and Estimation

The baseline characteristics of patients assigned to the three treatment groups included patient gender, diagnosis of TMD, body mass index (BMI), stress levels, anxiety levels, presence of systemic diseases, and signs and symptoms of TMD (headaches, joint sounds, and locked jaw), as are shown in Table 1. 

The age (*p*-value = 0.001) and drug intake (*p*-value = 0.039) had different effects among the three types of treatment, according to the bivariate analysis. 

The coefficients of the multivariate analysis are shown in Table 2. The RMS decreased significantly (*p*-value = 0.001) each week of the treatment: the RMS of GA decreased by 17%, that of GB decreased by 3%, and that of GC decreased by 5%. Muscle pain decreased each week of the treatment: that in GA decreased by 50%, that in GB decreased by 53%, and that in GC decreased by 71%. Joint pain decreased significantly (*p*-value = 0.041) with each week of the treatment. That in GA decreased by 79%, that in GB decreased by 72%, and that in GC decreased by 75%. Mouth opening increased significantly (*p*-value = 0.011) with each week of the treatment. In GA, it increased by 7%; in GB, it increased by 77%; and in GC, it increased by 94%. Joint sounds decreased each week of the treatment. In GA, it decreased by 19%; in GB, it decreased by 25%; and in GC, it decreased by 3%. 

The statistical differences among the groups are shown in Table 3 for muscle fatigue (*p*-value = 0.001), joint pain (*p*-value = 0.012), and muscle pain (*p*-value = 0.003). The comparison among the groups (Bonferroni) determined that a difference in muscle fatigue (*p*-value = 0.001), joint pain (*p*-value = 0.009), and muscle pain (*p*-value = 0.003) appeared between the occlusal splint and transcutaneous electrostimulation plus splint groups.

## 4. Discussion

In total, 91 patients were treated. Their average age was 31.2 years, 72% were women, and the predominant diagnosis of TMD was the onset of pain and headache.

The moderate and medium types of stress were the most predominant in the sample, compared with the level of anxiety, as the low level of anxiety was the most frequent. 

Management of temporomandibular disorders (TMDs) should reduce or eliminate pain and articulatory noise and restore normal jaw function. It is well established in the literature that most patients achieve good symptom relief with conservative measures, and it seems that almost every patient eventually improves over time regardless of the treatment they receive [28]—according to the natural history of the disease—if it is not progressive. However, it is a complex disorder that is shaped by many interacting factors that serve to maintain the disease [29,30,31].

The evaluation of the changes in variables across the treatment period showed that the RMS decreased significantly each week to a greater extent in GA, muscle pain decreased to a greater extent in GB, joint pain decreased to a greater extent in GA, mouth opening increased to a greater extent in GC, and joint sounds decreased to a greater extent in GB. 

The comparison among the groups determined that the transcutaneous electrostimulation therapy, as an adjuvant to the use of an occlusal splint, decreased muscle fatigue, muscle pain, and joint pain to a greater extent during each week of treatment, compared with the use of percutaneous therapy and a splint. 

The results of the present study show that the effects produced by the different types of treatments are elective, thus highlighting the importance of not only considering the clinical effect, but also of objectively evaluating the neuromuscular effect that occurs during the treatment. 

EMG is a very low-risk procedure, and complications are unusual. There is a low risk of hemorrhage, infection, and nerve injury when recording with needles, where a needle-shaped electrode is inserted [32].

The objective evaluation of masticatory function and frequent monitoring of neuromuscular activity through electromyography studies are the strengths of the present study. The implementation of this diagnostic tool provided quantitative information on masticatory function by using appropriate analyses to assess the electrical activity and muscle fatigue. 

Just a few studies have determined muscle strength by measuring the muscles’ electric current via the amplitude and frequency of the electromyogenic signal. The present study also evaluated muscle fatigue through multifractal analysis, which allowed for a more accurate characterization of this complex muscle process, providing a general evaluation of the masticatory function. The use of these tools counteracted the diagnostic difficulties that complicated the treatment plans, which is the main weakness of TMD studies [32,33,34].

Regarding other studies, these results showed the effects of the current approach, which involves the implementation of complementary techniques for the relief of the signs of temporomandibular disorders [35]. We agree with Macedo [36], who stated that a splint is effective for treating TMD. However, he questioned whether the use of a splint may limit the complete remission of symptoms, justifying the use of physical therapies as adjuvants in the treatment of TMD [37]. 

We found that electrostimulation therapies are therapeutic aids that produce analgesic effects and muscle relaxation. We agree with Conti [38] and Pierce [39] about the efficacy of splints, which explained why this therapy is the most common treatment modality applied in odontology for TMD and confirmed that this modality is the primary approach and is used as therapy to control the symptoms associated with TMD. The results showed that the use of a splint reduced muscle pain to a greater extent in less time, compared with the other types of treatment. 

Lundh [39] said that the muscular effects produced by the therapies varied, and highlighted that therapeutic success is affected by the duration and type of therapy.

The results reflect the difference in the clinical and muscular scope of each of the treatments. For example, percutaneous electrostimulation therapy decreased joint pain and neuromuscular electrical activity further (RMS). Transcutaneous electrostimulation therapy decreased muscle fatigue and the presence of joint sounds to a greater extent, whereas the use of an occlusal splint decreased muscle pain and increased mouth opening.

However, our results do not agree with the controlled studies of Barrero [40] and Ferreira [41,42], regarding the application of transcutaneous electrostimulation therapy in patients with severe symptoms of TMD. These authors reported a significant decrease in myofascial pain and muscle and joint sensitivity, and an increase in oral opening after four sessions of transcutaneous electrostimulation therapy. 

The effects observed with percutaneous electrostimulation are controversial, although we observed positive effects. We agree with Zhang [43] and Quiroz [23,44], who determined that the results depended on the point selected to be stimulated, the stimulus method used, and the duration of the stimulus [45,46].

The results of the present study have several implications for clinicians wishing to optimize the treatment of TMDs, including the diagnosis of TMDs, selection of the treatment, neuromuscular monitoring during treatment, and training in electrostimulation therapies for the treatment of TMD [47].

It would be interesting to know the clinical and muscular effect in patients with TMD treated with physical therapies in which other areas or other points are stimulated [23,44,45,46]. For future research, it is suggested to measure the effect on psychosocial factors (level of anxiety, stress, and/or depression) during treatment with the use of electrostimulation therapy [41].

## 5. Conclusions

Electrostimulation therapies represent significant added value as a complement to other conservative therapies such as splints, physiotherapy, and self-control.

Electrostimulation therapies optimize the treatment of TMD and reduce the main signs and symptoms in less time.

Transcutaneous electrostimulation therapy, as an adjuvant to the use of splints, reduced joint pain and muscle fatigue to a greater extent in less time. 

An evaluation of the muscle is crucial for diagnosis and during the treatment of TMD.

The implementation of electromyographic analysis for evaluating the muscle provides generalized information on masticatory function. 

## Figures and Tables

**Table 1:** Comparison of the demographic and clinical characteristics between the groups.

	Variable	Transcutaneous Electrostimulation and Occlusal Splint (n = 31)	Percutaneous Electrostimulation and Occlusal splint (n = 27)	Occlusal Splint (n = 33)	*p*-Value
Sex	Female	28	23	25	0.456 *
	Male	8	4	5	
Age	P50 (IQR)	42 (22)	30 (24)	23 (7)	0.001 +
TMD Diagnosis	Degenerative	5	3	10	0.405 *
	Painful	18	15	17	
	Intra-articular	7	8	6	
BMI	Low weight	1	2	1	0.096 *
	Normal	11	18	17	
	Overweight	13	4	6	
	Obesity	6	3	9	
Stress	Low	2	6	3	0.213 *
	Slight	12	12	16	
	Moderate	15	9	14	
	Severe	2	0	0	
Anxiety	Very low	14	21	20	0.069 *
	Moderate	17	5	12	
	Severe	0	1	1	
Systemic diseases	No	14	20	18	0.079 *
	Yes	17	7	15	
Drug intake	No	11	19	17	0.039 *
	Yes	19	8	16	
Headache	No	9	12	12	0.528 *
	Yes	21	15	21	
Joint sounds	No	2	3	2	0.739 *
	Yes	28	24	31	
Mouth opening	No	23	18	21	0.442 *
	Yes	7	10	12	

* x^2^ or Mann-Whitney U test (statistical model used to determine *p*-value); + Poisson regression (statistical model used to determine *p*-value).

**Table 2:** Changes in electromyographic and clinical variables over time during the treatment of TMD, multivariate models.

		RMS		Hurst		Joint Pain		Muscle Pain		Mouth Opening		Joint Sounds
	Time	Mean	Std. Dev.	Mean	Std. Dev.	Mean	Std. Dev.	Mean	Std. Dev.	Mean	Std. Dev.	Positive Cases
Splint	T0	211.305	112.350	0.090	0.044	4.091	2.740	4.394	2.900	42.818	8.520	30
	T1	199.108	109.820	0.097	0.068	3.424	3.130	4.364	1.980	43.545	7.770	30
	T2	165.294	93.950	0.094	0.047	3.818	2.770	3.424	2.440	43.970	7.580	30
	T3	205.206	93.110	0.079	0.067	2.909	2.630	2.394	2.190	45.455	6.500	30
	T4	163.345	93.440	0.079	0.067	1.970	2.660	1.545	1.470	44.758	6.370	25
	T5	155.992	89.720	0.076	0.059	1.121	2.140	1.182	1.870	45.848	5.780	25
Percutaneous	T0	204.359	181.550	0.095	0.036	4.963	3.500	4.667	3.120	39.889	9.970	26
	T1	189.746	143.570	0.108	0.075	4.481	3.340	4.259	3.180	43.185	9.410	25
	T2	73.202	43.340	0.102	0.041	3.556	3.550	4.185	3.250	43.481	7.990	26
	T3	197.035	55.110	0.099	0.056	2.630	2.550	3.148	3.530	46.704	6.990	25
	T4	87.320	36.620	0.086	0.035	2.667	3.150	2.815	3.180	45.370	7.020	20
	T5	77.350	135.140	0.082	0.036	2.667	3.030	2.630	3.230	46.963	5.610	19
Transcutaneous	T0	151.647	111.960	0.118	0.047	4.839	3.150	5.161	3.950	43.290	8.790	29
	T1	150.452	114.460	0.105	0.062	4.194	3.130	4.452	3.670	43.968	8.760	27
	T2	102.521	73.710	0.111	0.038	3.581	3.330	3.355	3.390	44.968	7.520	28
	T3	151.049	56.910	0.100	0.040	3.613	3.140	3.839	3.130	44.290	8.570	26
	T4	92.051	82.690	0.106	0.069	3.290	3.180	3.419	3.370	42.710	6.940	21
	T5	91.172	69.660	0.097	0.041	3.419	3.460	2.419	0.500	44.484	6.040	19
	Pr (>Chisq) by treatment	0.014		0.046		0.278		0.200		0.897		0.061
	Pr (>Chisq) by session for treatment	0.019		0.947		0.041		0.200		0.011		0.213

**Table 3:** Comparison of the changes among groups, estimated by analysis of variance (ANOVA).

Treatment	Summary		ANOVA	Bonferroni					
**RMS**	**Mean**	**Std. Dev.**	**Prob > F**	**Splint and ** **Transcutaneous**		**Splint and** **Percutaneous**		**Percutaneous and** **Transcutaneous**	
				**Dif p/Group**	***p*-Value**	**Dif p/Group**	***p*-Value**	**Dif p/Group**	***p*-Value**
Splint	179.980	101.020	0.6280					85.377	1.000
Percutaneous	134.550	130.430				−45.430	1.000		
Transcutaneous	219.930	151.740		39.946	1				
**Hurst**	**Mean**	**Std. Dev.**	**Prob > F**	**Splint and transcutaneous**		**Splint and percutaneous**		**Percutaneous and transcutaneous**	
				**Dif p/group**	***p*** **-value**	**Dif p/group**	***p*** **-value**	**Dif p/group**	***p*** **-value**
Splint	0.085	0.059	0.0013					0.011	0.194
Percutaneous	0.095	0.049				0.011	0.290		
Transcutaneous	0.106	0.051		0.020	0.001				
**Joint pain**	**Mean**	**Std. Dev.**	**Prob > F**	**Splint and transcutaneous**		**Splint and percutaneous**		**Percutaneous and transcutaneous**	
				**Dif p/group**	***p*** **-value**	**Dif p/group**	***p*** **-value**	**Dif p/group**	***p*** **-value**
Splint	2.790	2.845	0.012					0.450	0.443
Percutaneous	3.222	3.312				0.431	0.480		
Transcutaneous	3.672	3.231		0.882	0.009				
**Muscular pain**	**Mean**	**Std. Dev.**	**Prob > F**	**Splint and transcutaneous**		**Splint and percutaneous**		**Percutaneous and transcutaneous**	
				**Dif p/group**	***p*** **-value**	**Dif p/group**	***p*** **-value**	**Dif p/group**	***p*** **-value**
Splint	2.629	2.489	0.0038					0.351	0.759
Percutaneous	3.248	3.277				0.618	0.125		
Transcutaneous	3.600	3.443		0.970	0.003				
**Mouth opening**	**Mean**	**Std. Dev.**	**Prob > F**	**Splint and transcutaneous**		**Splint and percutaneous**		**Percutaneous and transcutaneous**	
				**Dif p/group**	***p*** **-value**	**Dif p/group**	***p*** **-value**	**Dif p/group**	***p*** **-value**
Splint	45	7.015	0.8100					0.487	1.000
Percutaneous	45	45				−0.221	1.000		
Transcutaneous	45	8		0.256	1.000				

## Data Availability

Follow-up of this project and data supporting the reported results generated during this study are available under Protocol Registration and Approval at Clinical Trials.gov: Protocol ID: CIE/0508/02/2020 and ClinicalTrials.gov: NCT04704778.
